# Software-based noise reduction in cranial magnetic resonance imaging: Influence on image quality

**DOI:** 10.1371/journal.pone.0206196

**Published:** 2018-11-01

**Authors:** Philipp Fuelkell, Soenke Langner, Nele Friedrich, Marie-Luise Kromrey, Christoph G. Radosa, Ivan Platzek, Birger Mensel, Jens-Peter Kühn

**Affiliations:** 1 Department of Diagnostic Radiology and Neuroradiology, University Medicine Greifswald, Greifswald, Germany; 2 Department of Radiology, University Medicine Rostock, Rostock, Germany; 3 Department of Laboratory Medicine, University Medicine Greifswald, Greifswald, Germany; 4 Clinic and Policlinic for Radiology and Interventional Radiology, Carl-Gustav-Carus University, Dresden, Germany; Johns Hopkins School of Medicine, UNITED STATES

## Abstract

**Objectives:**

To investigate acoustic noise reduction, image quality and white matter lesion detection rates of cranial magnetic resonance imaging (MRI) scans acquired with and without sequence-based acoustic noise reduction software.

**Material and methods:**

Thirty-one patients, including 18 men and 13 women, with a mean age of 58.3±14.5 years underwent cranial MRI. A fluid-attenuated inversion recovery (FLAIR) sequence was acquired with and without acoustic noise reduction using the Quiet Suite (QS) software (Siemens Healthcare). During data acquisition, peak sound pressure levels were measured with a sound level meter (Testo, Typ 815). In addition, two observers assessed subjective image quality for both sequences using a five-point scale (1 very good—5 inadequate). Signal-to-noise ratio (SNR) was measured for both sequences in the following regions: white matter, gray matter, and cerebrospinal fluid. Furthermore, lesion detection rates in white matter pathologies were evaluated by two observers for both sequences. Acoustic noise, image quality including SNR and white matter lesion detection rates were compared using the Mann-Whitney-U-test.

**Results:**

Peak sound pressure levels were slightly but significantly reduced using QS, *P*≤0.017. Effective sound pressure, measured in Pascal, was decreased by 19.7%. There was no significant difference in subjective image quality between FLAIR sequences acquired without/with QS: observer 1: 2.03/2.07, *P* = 0.730; observer 2: 1.98/2.10, *P* = 0.362. In addition, SNR was significantly increased in white matter, *P*≤0.001, and gray matter, *P* = 0.006, using QS. The lesion detection rates did not decline utilizing QS: observer 1: *P* = 0.944 observer 2: *P* = 0.952.

**Conclusions:**

Sequence-based noise reduction software such as QS can significantly reduce peak sound pressure levels, without a loss of subjective image quality and increase SNR at constant lesion detection rates.

## Introduction

Overexposure to excessive acoustic noise may result in temporary or permanent noise induced hearing loss (NIHL). [1] MRI acquisition at 1.5 Tesla produces acoustic noise levels of nearly 120 dB(A). Despite advanced hearing protection, these noise levels may cause serious acoustic inner ear damage and preclude certain patients, such as those with tinnitus, from having an MRI examination. [2,3] Therefore lower MRI decibel levels are of considerable interest in terms of patient safety precautions. Moreover, acoustic-noise-related stress is one of the most common complaints of patients undergoing magnetic resonance imaging (MRI). In addition, high acoustic stress levels during MR examinations may be a major cause of motion artifacts, leading to poor image quality and loss of diagnostic information. Lower MRI decibel levels could reduce anxiety, which is especially important in children and patients with dementia or claustrophobia, and thus improve patient comfort and satisfaction in general. [4,5]

Most healthcare providers make a huge effort and continuously upgrade their medical equipment to reduce acoustic noise. The gradient coil system is the most important source of acoustic noise. [5] Authors suggest that sequence-based approaches such as changes in sequence parameters or sequence type (e.g., spin echo versus gradient echo sequences) are considerably more expedient than hardware replacement for the purpose of acoustic noise reduction. [5] Various manufacturers therefore turn to sequence-based innovation management to decrease acoustic noise.

Several software products are currently available that incorporate acoustic noise management during MR imaging. For instance Silenz (GE Healthcare, Milwaukee, Wisconsin, USA) features an acoustic noise dampening scan software that achieves significant acoustic noise reduction by using a 3D gradient-echo imaging technique with a very short TE and low flip angles [6]. With Pianissimo (Toshiba, Tusin, CA, USA), significant acoustic noise reduction is accomplished using an acoustic noise-minimizing technology that links vacuum-sealed gradient coils with software-optimized silent pulse sequences. [6] Another approach is called Quiet Suite (QS) (Siemens Healthcare, Erlangen, Germany). It is an MRI sound-reducing technology that decisively lowers sound pressure levels (SPLs) during acquisition of conventional MRI sequences without any hardware modifications. Rapidly switching gradients, producing mechanical vibrations, are the source of excessive SPLs in the course of an MRI examination. QS dampens the induced scanner vibrations by optimizing and smoothing the gradient trajectory. QS implements an elaborate summation of gradients and reduction of slew rates while maintaining timing parameters in a reasonable range. [7,8]

QS as an alternative approach should lead to an effective reduction of acoustic noise without loss of diagnostic information. [9] However, to the best of our knowledge, there is limited literature on systematic comparisons of reduction of acoustic noise levels and image quality, including a signal-to-noise ratio (SNR) analysis, for image acquisition performed in a clinical setting with and without the QS acoustic noise reduction software.

Therefore, the aim of our study was to investigate acoustic noise reduction, image quality and white matter lesion detection rate of cranial MRI scans acquired with and without sequence-based acoustic noise reduction software.

## Material and methods

This study was approved by the local ethics committee of the University of Greifswald (BB 061/14). All subjects gave written informed consent separately for study inclusion and for cranial MRI.

### Study population

Between August 2014 and January 2015, patients who underwent cranial MRI for workup of intracranial pathology were randomized and prospectively enrolled in this study.

Inclusion criteria for this study were age between 18 and 90 years and completion of the clinically indicated MRI examination. Exclusion criteria were contraindications to MRI such as pacemakers, non-MRI-compatible implants, and pregnancy. Patients who were not capable of giving proper written informed consent were also excluded from the study.

Thirty-one patients, including 18 men and 13 women, were enrolled in this study. They had a mean age of 58.3±14.5 years. Clinical indications for cranial MRI ranged from outpatient elective diagnostic imaging (n = 25), e.g., screening for metastasis and multiple sclerosis, to emergency diagnostic imaging (n = 6), e.g., for acute brain infarction.

### MR imaging

MR examinations were performed on a 1.5-Tesla MR scanner (Siemens Aera, Siemens Healthcare, Erlangen, Germany) using a 20-channel head coil. Acoustic noise reduction and image quality of QS were evaluated in fluid-attenuated inversion recovery (FLAIR) sequences, which are clinically important to confirm and rule out intracranial pathologies, such as demyelinating lesions, tumors, infarcts, and bleedings. Therefore, the study protocol included FLAIR sequences acquired without QS and with QS. All other imaging parameters were identical for both sequences: TR/TE: 9000/82ms; inversion time: 2500ms; flip angle: 150°; bandwidth: 190Hz/px; image resolution: 210x320; field of view: 230mm; slices: 24; slice thickness: 5mm; echo train length: 16. Both sequences were acquired without parallel imaging. The sequences were performed without any image filters or pre-scan normalizing. Total acquisition time was 4:32 min for both sequences. Acquisition of the two FLAIR sequences with- and without QS was followed by additional pulse sequences as clinically required in each patient.

### Quiet Suite algorithm

QS utilizes an algorithm that works irrespective of sequence programming and processing. The algorithm is able to discriminate between certain sequence components, dividing the sequence into two major gradient categories, k-gradients (“keep” gradients) and c-gradients (“change” gradients).

For instance, readout gradient and slice selection gradient lobes rank among k-gradients. The QS algorithm does only affect and change c-gradients. A spline interpolation method is then used to create a smooth gradient form between consecutive k-gradients at constant timing parameters. The k-gradient moment remains uncompromised. This allows for slew rate reduction and restraint of harmonic excitation within the gradient system. [8]

### Sound pressure & sound pressure levels

Sound pressure refers to the pressure fluctuations of air caused by sound propagation. The SI unit of sound pressure is pascal (Pa). Sound pressure, specified p, is definded by [8,10]

$$P_{total}=P_{static}+P_{dynamic}$$

The local ambient pressure is overlaid by dynamic pressure (change of pressure).

Whereas sound pressure level (SPL) describes a logarithmic measure of the effective pressure of a sound wave in relation to a reference value. SPL, measured in dB, denoted L, is defined by [8,10]

$$L=10\times \log_{10}\left(\frac{p^{2}}{p_{0}{}^{2}}\right)\;dB=20\times \log_{10}\left(\frac{p}{p_{0}}\right)\;dB$$

### Measurement of peak sound pressure levels

Peak SPLs were measured throughout acquisition using a sound level meter (Testo, Typ 815; ISO9001 certified; Testo GmbH & Co, Lenskirch, Germany). All patients were imaged with the device positioned inside the examination room but outside the bore, two meters away from the MRI magnet front panel, with the microphone pointing towards it. The Testo Typ 815 utilizes a precision electret-condensor microphone sensor at 1/2inch. The device allows for three different types of weighting. For the purpose of our study the Testo 815 DIN/IEC 651 was set with the most common weighting: A. The measuring range was 30 to 130 db(A). A frequency range of 31,5 Hz to 8 kHz at a reference frequency of 1000 Hz was selected. A time setting of 125ms (fast setting) was applied. The device was zeroized and calibrated in an appropriate fashion as outlined in the technical manual before each measuring cycle.

Prior to use in patient examinations, the sound level meter was tested in a phantom experiment to identify peak SPL when acquiring the sequences with exactly the same imaging parameters as described above to ensure scan reliability and check for possible magnetic-field-related malfunctioning of the sound level meter. Therefore, a set of four test scans with an empty bore was acquired using a standard head phantom.

For acoustic noise comparison of SPL peak values we calculated the parameters as follows:

SPL difference among sequences without QS (A) and with QS (B):

ΔL=A−B

Assessment of sound pressure factor difference:

$$\Delta SP=10^{\Delta L/20}$$

Sound pressure [8,10,11]:

$$p=p_{0}\times 10^{L_{p}/10}Pa\;\left(p_{0}=20\mu Pa\right)$$

### Data analysis

Image quality of both FLAIR sequences was independently compared and rated by one board-certified radiologist with 11 years of experience in MR imaging and one resident in radiology with one year of experience in MR imaging. Imaging analysis was performed using a PACS workstation (Impax version 6.1, AGFA, Belgium) allowing simultaneous display of both sequences. The two readers graded image quality on a five-point scale without knowing whether the noise reduction software was used: 1 = very good; 2 = good; 3 = satisfying; 4 = diagnostic, and 5 = inadequate. Scores of 1–3 were considered to indicate adequate diagnostic image quality.

SNR analysis was performed for both sequences using Osirix version 5.0 (Pixmeo, Bernex Switzerland) by an observer who was not involved in subjective image analysis. For this reason, signal intensities (SI) were assessed in pairs of three regions of interest (ROI) placed in both hemispheres in the frontal horn of the lateral ventricle, and the temporoparietal gray matter and frontal white matter. In addition, another two pairs of ROIs were placed outside the cranial scan to determine the standard deviation of background noise. While the size of the ROI for measurement of background noise was the same for all individuals, the size of the other ROIs was adjusted individually but kept constant intraindividually and copied between the two sequences. In case of misplacement between both sequences due to head movement, the ROI was replaced manually to ensure reproducibility.

The fixed ROI size for all four background noise areas was 3.030cm^2^. The ROI size for measurement of SI was chosen as large as possible. The average ROI size for SI of white matter was 0.211 cm^2^. The average ROI size for SI of gray matter was 0.093 cm^2^. The average ROI size for SI of cerebrospinal fluid was 0.201 cm^2^.

Mean signal intensities in the defined ROIs and the standard deviation of background noise were measured. SNR was calculated as follows:

$$SNR=\frac{mean\;signal\;intensity}{standard\;deviation\;of\;background\;noise}$$

In addition, white matter lesions of both sequences, with and without QS, were independently evaluated by one board-certified radiologist with 15 years of training in MR imaging and a senior resident with 4 years of experience in MR imaging. The two readers reported the total amount of detected white matter lesions (defined as focal lesions with high signal intensity in FLAIR imaging) for each sequence in a randomized, two blinded data setting.

### Statistical analysis

Continuous variables are provided as mean ± standard deviation and additionally for peak SPL, as range of minimum and maximum values.

The Mann-Whitney-U test was used to evaluate differences in acoustic noise levels, subjective image quality, SNR, and WML detection rates for sequence acquisition without QS compared to acquisition with QS.

In addition, peak SPLs for sequences with QS and without QS were correlated with patients’ body mass index (BMI) to investigate whether a patient-related factor may influence the generation of acoustic noise during image acquisition. Therefore, the study population was categorized into three subgroups according to their current BMI status: healthy (BMI ≤ 24,9 kg/m^2^), overweight (25 kg/m^2^ ≤ BMI ≥ 29,9 kg/m^2^), obese (BMI ≥ 30 kg/m^2^). The data had been analyzed using the Kruskal-Wallis test to evaluate differences between subgroups.

The level of significance was defined as a p-value ≤ 0.05.

## Results

MRI was successfully performed in all patients without and with the QS acoustic noise reduction software.

In the phantom, SPL at baseline (i.e., before image acquisition started) was 52.1±0.1dB(A). Acoustic noise measurement in phantoms demonstrated slightly lower SPL values for sequences acquired with QS compared to the series without QS (75.1±1.0dB(A) versus 76.5±0.5dB(A), *P* ≤ 0.012).

**Fig 1:**
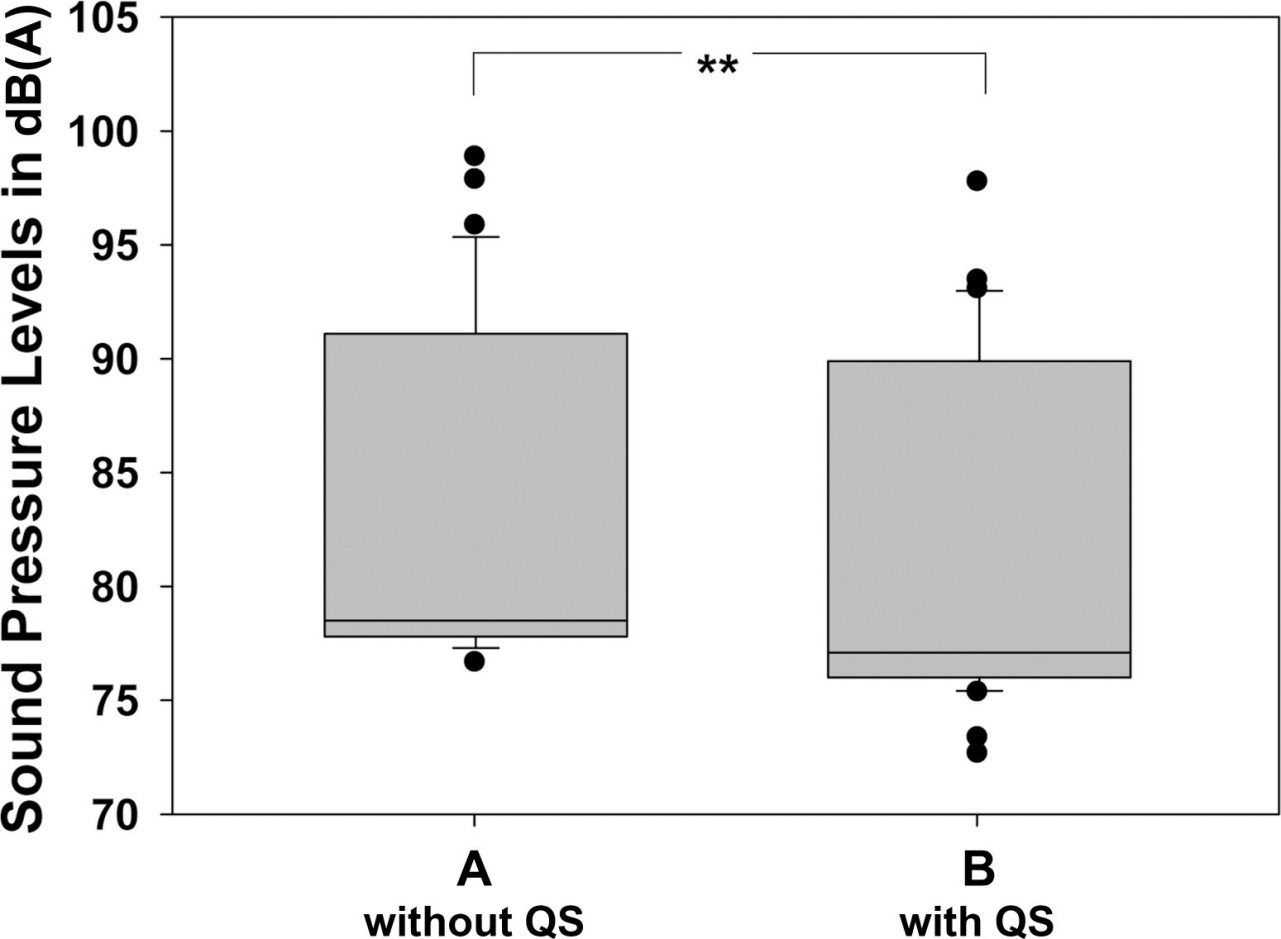
Mean peak sound pressure level in dB(A) for the two FLAIR sequences. A: acquired without QS (QuietSuite) acoustic noise reduction software; B: acquired with QS. There was a slight but statistically significant noise reduction with use of QS. ** *P* ≤ 0.001.

In patients, SPL at baseline was 52.9±1.0dB(A). **Fig 1** presents boxplots of peak SPLs measured without and with the use of QS. In accordance to the phantom data, there was a slight, but significant difference in SPL peak values in patients between the sequence without QS and the sequence with QS (83.5±7.3dB(A) versus 81.5±7.5dB(A), *P* ≤ 0.017). QS reduced effective sound pressure, measured in Pascal, by 19.7%. Both, peak SPL and sound pressure measurements are shown in **Table 1**. Nevertheless, there was a high range of peak SPL values for sequences without QS (minimum 76.7 dB(A), maximum 98.9 dB(A)) and also for acquisitions with QS (minimum 72.7 dB(A), maximum 97.8 dB(A)).

**Table 1:** QuietSuite impact on acoustic noise.

	mean peak SPL	ΔL = A-B	Sound pressure	ΔSP(factor) = 10Δ^L/20^	Reduction of SP
Without QS (A)	83,45 db(A)	1,91 db(A)	0,2975 Pa	1,246	19,7%
With QS (B)	81,54 db(A)		0,2388 Pa		

We revealed no differences between peak SPL and groups of BMI (healthy, overweight, obese in sequence with QS (P = 0.353) and without OS (*P* = 0.194).

**Fig 2:**
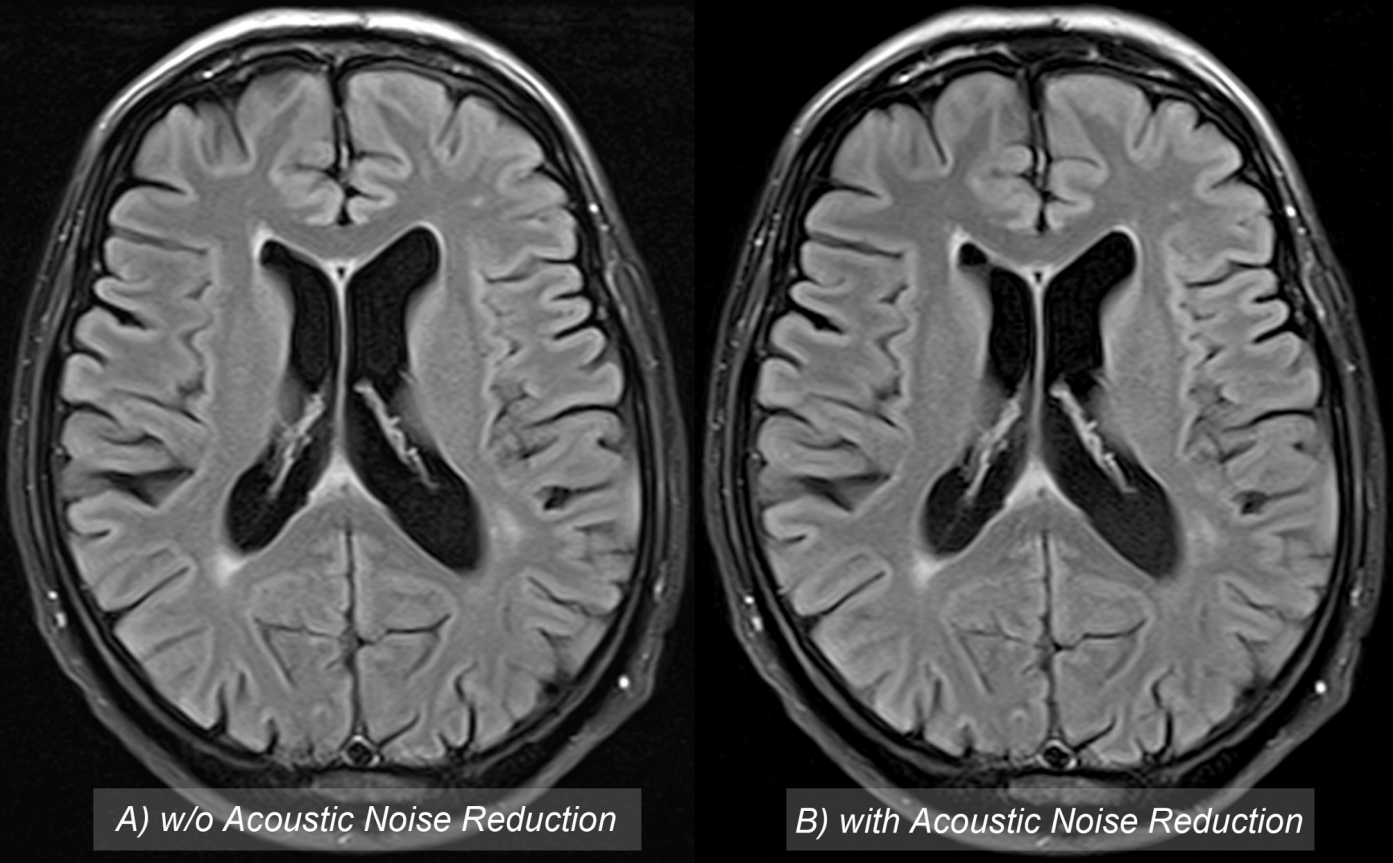
65-year-old male patient who underwent cranial MRI to screen for metastasis. The examples illustrate the quality of FLAIR images acquired without (left) and with (right) acoustic noise reduction software. There was no difference in subjective image quality, and both sequences were of diagnostic quality.

**Fig 3:**
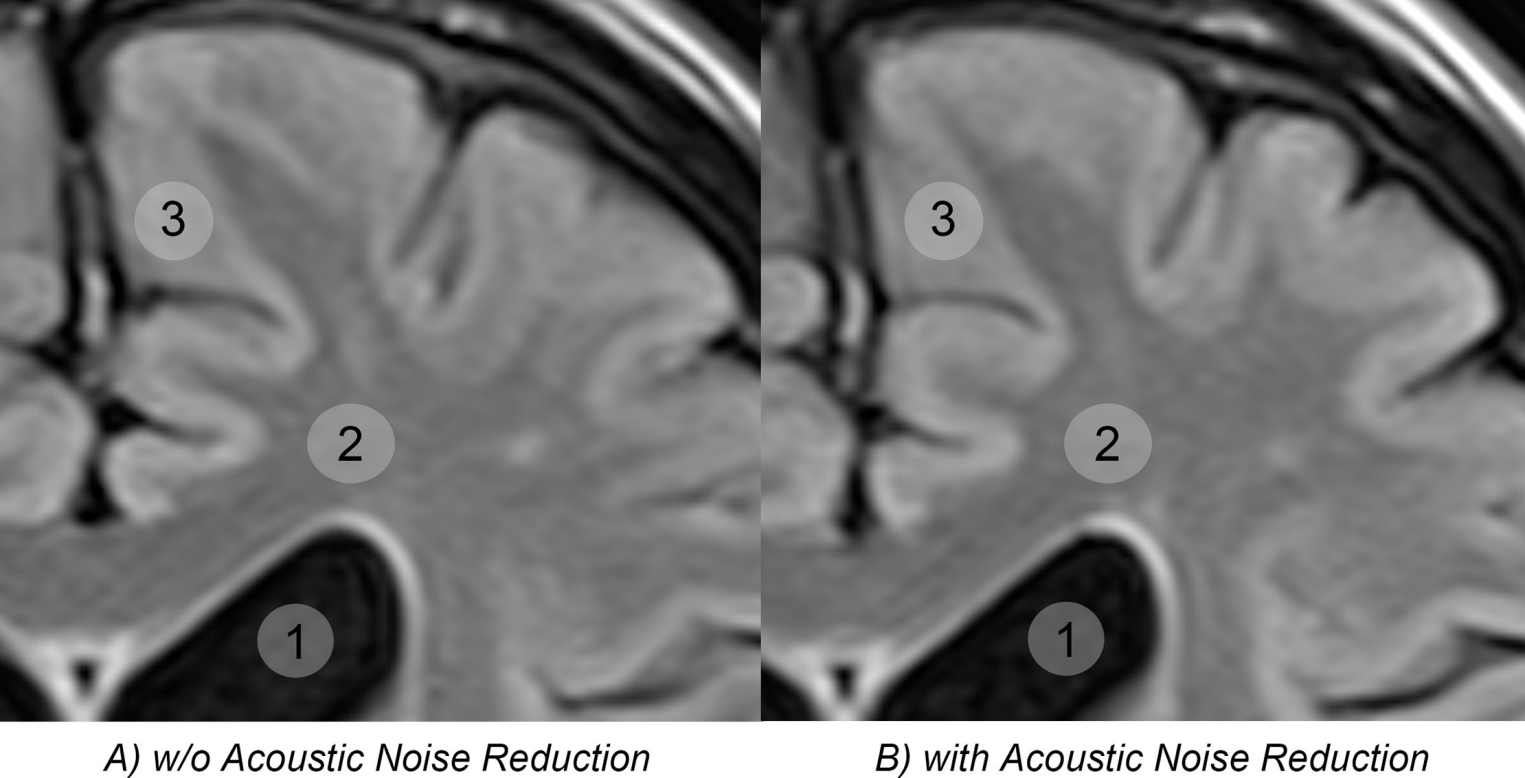
Image quality. Unchanged image quality between sequences acquired with and without acoustic noise reduction software is also confirmed in these magnified details. The circles are regions of interests (ROIs) placed in CSF (1), gray matter (2), and white matter (3) for quantative signal-to-noise analysis.

**Figs 2 and 3** present examples of brain image quality with and without QS. Subjective image analysis yielded comparable results for both sequences without significant differences in grading between observer 1 (*P* = 0.730) and observer 2 (*P* = 0.362). Observer 1 assigned a grade point average (GPA) of 2.03 using QS and 2.07 without QS. “Good” or “very good” ratings were assigned to 93.6% of the images acquired with QS versus 87.10% of the images acquired without QS. For observer 2, the scores revealed a GPA of 1.97 using QS and 2.10 for sequences without QS. Observer 2 rated 93.6% of sequences acquired with QS as „good”or „very good”compared to 87.1% of sequences acquired without QS.

**Fig 4:**
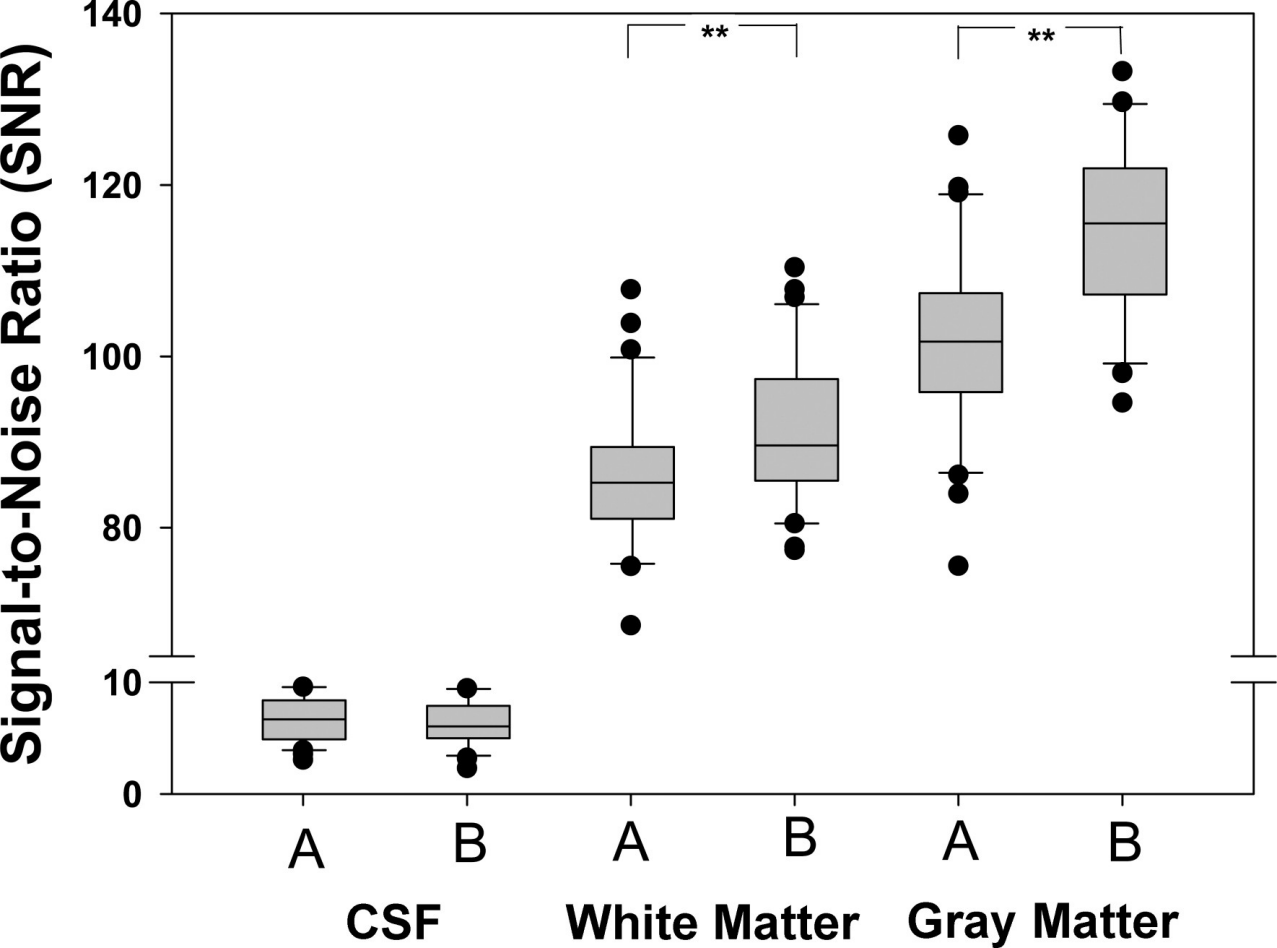
Signal-to-noise ratio (SNR) analysis in three regions of interest. CSF, white matter, and gray matter. Sequence A was acquired without acoustic noise reduction software and sequence B with the acoustic noise reduction software. Use of the noise reduction software significantly increased SNR. ** *P* ≤ 0.001.

**Table 2** demonstrates the mean signal intensity and the standard deviation of the background noise for grey matter, white matter and liquor cerebrospinalis for both sequences. **Fig 4** separately displays mean SNR for white matter, gray matter and cerebrospinal fluid (CSF) without and with the use of acoustic noise reduction software. As expected, SNR analysis for CSF (*P* = 0.235) did not show a significant difference, yielding a mean SNR of 6.7±2.0 with QS and of 6.3±2.1 without QS. The SNR scores for white matter indicated a significant increase in SNR for the use of QS (101.8±11.5 versus 114.9±10.1, *P* < 0.001). There was also a significant difference in SNR for gray matter between both sequences (85.5±9.5 with QS versus 91.4±8.6 without QS, *P* < 0.006).

**Table 2:** Changes in background noise (SD), signal intensity (SI) and signal-to-noise ratio (SNR) according to the use of QuietSuite.

	Without QS	With QS	*P*
SD Background Noise	3.71 ± 0.38	3.36 ± 0.21	**<0,001**
SI liquor cerebrospinalis	24.60 ± 7.21	20.93 ± 6.48	**0.039**
SI white matter	374.97 ± 37.10	384.17 ± 24.98	0.398
SI gray matter	314.57 ± 28.89	306.10 ± 29.99	0.131
SNR liquor cerebrospinalis	6.68 ± 2.01	6.30 ± 2.11	0.235
SNR white matter	101.81 ± 11.52	114.92 ± 10.09	**<0,001**
SNR gray matter	85.46 ± 9.47	91.36 ± 8.59	**0.006**

Values presented as mean ± standard deviation.

The lesion detection rate evaluation for both readers yielded no significant differences with and without QS. The comparison of WML detection rates between the regular FLAIR sequence and the QS based sequence showed no significant difference for reader 1 (*P =* 0.9442). Likewise, the WML detection rates by reader 2 did not significantly differ between both sequences. (*P* = 0.95216). Overall consistency for white matter lesion detection rates between the two sequences was 87.1% for both readers. QS based FLAIR sequences revealed a lower WML rate in 6.45% of all cases for reader 1 and reader 2 respectively. The total rate of white matter lesions for each sequence was highly dependent on the underlying pathology. For instance, WML detection rates in patients with demyelinating lesions in multiple sclerosis was reported as high as n = 84 without QS, n = 83 with QS by reader 1. Whereas reader 2 detected n = 79 for both sequences. WML detection rates in certain pathologies, such as acute brain infarction, was commonly limited to unifocal white matter lesions only (n = 1) for both sequences.

## Discussion

In this study, we investigated the impact of the QS acoustic noise reduction software on SPL and image quality in routine clinical FLAIR imaging. The use of QS significantly reduced the SPL at no loss of subjective image quality. QS resulted in a significant increase in SNR. Detection rates of white matter lesion were not compromised using QS.

Recent studies have investigated different approaches to acoustic noise reduction in MRI such as optimization of gradient hardware by means of vacuum chamber-enclosed gradient coil systems [12–14] or mechanic gradient field rotation designs [15]. Furthermore, other gradient waveform redesigns such as low-pass filtering gradient systems [16] or composite waveforms with parallel imaging [17,18] have been proposed. Ireland et al. investigated the acoustic performance of a novel acoustically quiet coil in a neonatal patient collective. The results showed a SPL reduction of 9 dB(A) on average due to the coil’s sound abating framework design. [19] Heavier gradient coils as well as floor-mounted gradient coil structures have proven to achieve acoustic noise reduction in MR imaging. [12] Mansfield et al. showed that gradient coil structure re-design, such as a split plate arrangement may optimize acoustic performance. [20] However, the feasibility of hardware—related noise cancelling approaches is often limited due to additional costs. Our results confirm that sequence-based approaches to acoustic noise reduction, such as QS, represent a viable alternative to expensive hardware solutions.

As of today, various research projects have been carried out to investigate the effects of sequence-based denoising techniques on MR image quality. For instance, Ott et al. showed a substantial acoustic noise decrease of up to 20 dB(A) at preserved image quality using an optimized diffusion-weighted imaging technique at 1.5 T and 3T. [21] The comparison of conventional T_2_ PROPELLER (periodically rotated overlapping parallel lines with enhanced reconstruction) and T_2_ FLAIR with quiet T_2_ PROPELLER and quiet T_2_ FLAIR sequences in a routine practice setting has also shown to effectively decrease SPLs (up to 28,5 dB(A)) while preserving image quality at slight scan time extension. [22] Fischer et al. presented a significant SPL reduction for a quiet optimized standard liver imaging protocol, consisting of TSE and gradient echo (GRE) sequences. SNR and CNR assessment confirmed no loss of image characteristics. [23]

Similar results were demonstrated by Alibek et al. and Ida et al. who investigated the performance of 3D T_1_ weighted inaudible MR sequences in day-to-day routine practice. [6]

Hybrid sequences such as PETRA (point-wise encoding time reduction with radial acquisition) achieve ultra short TE through combining single point imaging with radial projection imaging. Subsequently PETRA is not as dependent on gradient switching times and therefore less prone to eddy-currents disturbance and time delays. [24]

Most recently, these nearly inaudible scanning techniques have become commercially available. [6,7,25] Still, further comparative research with conventional scan methods is needed prior to commonplace clinical use.

Whereas image quality assessment was at the center of the above mentioned reports, SPL peak levels were not. Our results provide concordant data with recently published literature in terms of image quality preservation. The SPL analysis confirms noise reduction of acoustic peak measures, yet is not quite comparable to previous data from abovementioned research or the data provided by the vendor as we investigated peak SPL levels instead of mean SPL. In our opinion, the maximum SPL may be more crucial with regard to noise induced temporary or permanent hearing loss although the use of mean SPL is common practice. Especially high level transients seem to increase the hazard of NIHL. [26,27] Acoustic overexposures could contribute to tinnitus, hyperacusis and other perceptual anomalies commonly associated with inner ear damage. [27]

The overall reduction of the effective sound pressure in our patient data of 19.7% must be appraised critically. While we found an absolute decrease of 1.9 dB(A) in maximum peak sound level in patients—a small yet significant difference–the benefit to the patient in terms of minimized NIHL hazard or acoustic noise-related discomfort appears to be rather poor.

Image acquisition and SPL measurements were conducted under standardized conditions. Our findings show that utilizing QS does have a noise reduction impact on peak SPL levels. We postulate that there are various parameters that have a significant impact on SPL overall. Acoustic noise in MRI is closely linked to the gradient trajectory. [7,8] The type of pulse sequence as well as other imaging parameters such as TR, TE and FOV also influence the gradient trajectory to variable degrees. [28,29] Therefore, optimizations of these scan parameters may further decrease SPL during MR imaging. Ott et al. proposed five specific sequence parameter modification strategies to achieve overall SPL reduction of 16,8 dB(A) in T_1_- weighted and proton-density weighted turbo spin echo (TSE) acquisitions at no trade of image quality. [30]

Interestingly, we found a relatively wide variation in peak SPLs between patients of approximately 25 dB(A). Kruskal-Wallis analysis showed no significant correlation between peak SPL and patients’ body mass index. Other patient-related factors may include clothing or body temperature. More studies are necessary to explore connections between peak SPL and other patient-related factors.

Although gradient coil modifications may alter image quality, our findings showed no significant difference in image quality scores assigned by either reader. It was our objective to evaluate whether utilizing Quiet Suite does cause changes in SNR. Our findings demonstrate that SNR was affected. We found an increased SNR in brain tissue such as gray matter and white matter using QS. According to our data SNR increase was due to an increased signal intensity as well as a slightly decreased background noise. This observation could be related to algorithmic programming of the software. Parameter modifications that are most likely accountable for SNR increase involve variable encoding time, HF refocusing pulse duration, HF pulse duration, readout bandwidth and slice selection setting. [5,8,22]

The impact of sequence-based noise reduction algorithms on diagnostic accuracy is of particular interest in clinical routine practice. Therefore, we investigated a possible difference in detection of MR pathologies between standard and noise-reduced pulse sequences. Flair sequences yield excellent information on subtle changes at myelinated nerve tissue such as white matter. Thus, we provided an analysis of white matter lesion detection rates by two experienced readers. Our data showed that the detection rates of white matter lesions are the same for both sequences with and without QS. According to our results, denoising software techniques, such as QS, have the potential of replacing the regular pulse sequences in daily routine practice.

Our study has several limitations. We investigated an inhomogeneous study population. All MRI examinations were performed under routine clinical conditions, which attests to the study’s robustness. Another limitation is the study’s standardized design and that we only tested FLAIR sequences. However, we chose the FLAIR sequence technique because it is an important MRI sequence for detecting and ruling out pathology in the brain. In addition, QS is also available for other types of sequences such as T_2_-weighted turbo spin echo sequences, susceptibility-weighted imaging (SWI), or fast 3D T_1_-weighted gradient echo sequences. As mentioned above, different results might be obtained by using a combination of QS and other sequence techniques or sequence parameters. Further prospective studies are necessary to explore these aspects in greater detail. Another limitation of our study is that we only measured peak SPL instead of mean SPL and that we did not differentiate the spectra of frequencies over the entire acquisition time. Lastly, this study focused on image quality/SNR in healthy brain tissue and did not consider pathologic brain areas. For that reason we did not implement an additional CNR analysis. Admittedly, drawing ROIs for SNR analysis is a method with limited accuracy and may not produce results consistent with expectations in the analyzed protocols. [31] Moreover, SNR analysis might be prone to limited ROI selection and ROI size. [32] White matter lesion analysis was performed regardless of the underlying pathology e.g. demyelinating lesions in multiple sclerosis, subarachnoid haemorrhage or other leptomeningeal diseases as for instances meningitis. Diagnostic accuracy while utilizing noise-reduced pulse sequences has to be evaluated in further prospective studies in a larger patient cohort.

In conclusion, noise reduction software such as QS slightly reduces sound pressure levels while maintaining image quality using a standardized FLAIR sequence protocol. In addition, QS leads to an increase in SNR for cranial MRI acquisitions. Quiet FLAIR protocols may help to reduce the acoustic hazard of MR imaging at no trade of diagnostic accuracy and should be considered for routine clinical use.

## Supporting information

S1 File Dataset. QS_study_data.
(XLSX)

## Abbreviations

CSF: cerebrospinal fluid
dB: decibel
EPNL: effective perceived noise level
FLAIR: fluid-attenuated inversion recovery
GPA: grade point average
MRI: magnetic resonance imaging
PACS: picture archiving and communication system
QS: QuietSuite
ROI: region of interest
SPL: sound pressure level
SNR: signal-to-noise ratio
SI: signal intensity
NIHL: noise induced hearing loss
GRE: gradient echo
TSE: turbo spin echo
PROPELLER: periodically rotated overlapping parallel lines with enhanced reconstruction
WML: white matter lesion

## References

[1] Brummett RE, Talbot JM, Charuhas P. Potential hearing loss resulting from MR imaging. Radiology. 1988;169:539–40. 10.1148/radiology.169.2.3175004 3175004

[2] Price DL, De Wilde JP, Papadaki AM, Curran JS, Kitney RI. Investigation of acoustic noise on 15 MRI scanners from 0.2 T to 3 T. J Magn Reson Imaging. 2001;13:288–93. 11169836 10.1002/1522-2586(200102)13:2<288::aid-jmri1041>3.0.co;2-p

[3] Jin C, Li H, Li X, Wang M, Liu C, Guo J, et al Temporary Hearing Threshold Shift in Healthy Volunteers with Hearing Protection Caused by Acoustic Noise Exposure during 3-T Multisequence MR Neuroimaging. Radiology. Radiological Society of North America; 2017;286:602–8. 10.1148/radiol.2017161622 28813235

[4] Quirk ME, Letendre AJ, Ciottone RA, Lingley JF. Evaluation of three psychologic interventions to reduce anxiety during MR imaging. Radiology. 1989;173:759–62. 10.1148/radiology.173.3.2682775 2682775

[5] McJury M, Shellock FG. Auditory noise associated with MR procedures: a review. J Magn Reson Imaging. 2000;12:37–45. 10931563 10.1002/1522-2586(200007)12:1<37::aid-jmri5>3.0.co;2-i

[6] Alibek S, Vogel M, Sun W, Winkler D, Baker CA, Burke M, et al Acoustic noise reduction in MRI using Silent Scan: an initial experience. Diagn Interv Radiol. 2014;20:360–3. 10.5152/dir.2014.13458 24808439 PMC4463276

[7] Heismann B, Ott M, Grodzki D. Sequence-based acoustic noise reduction of clinical MRI scans. Magn Reson Med. 2015;73:1104–9. 10.1002/mrm.25229 24889327

[8] Ott M. Acoustic noise reduced MRI. 2015.

[9] Pierre EY, Grodzki D, Heismann B, Aandal G, Gulani V. Making MRI Scanning Quieter: Optimized TSE Sequences with Parallel Imaging. http://mri-q.com/uploads/3/4/5/7/34572113/quietx_mri-life-design-quiet-suite-magnetom-flash-55-pierre-01340944.pdf

[10] Hoiting GJ. Measuring MRI noise. 2005.

[11] Corcuera-Solano I, Doshi A, Pawha PS, Gui D, Gaddipati A, Tanenbaum L. Quiet PROPELLER MRI Techniques Match the Quality of Conventional PROPELLER Brain Imaging Techniques. American Journal of Neuroradiology. American Journal of Neuroradiology; 2015;36:1124–7. 10.3174/ajnr.A4235 25678482 PMC8013025

[12] Katsunuma A, Takamori H, Sakakura Y, Hamamura Y, Ogo Y, Katayama R. Quiet MRI with novel acoustic noise reduction. MAGMA. 2002;13:139–44. 11755088 10.1007/BF02678588

[13] Edelstein WA, Kidane TK, Taracila V, Baig TN, Eagan TP, Cheng Y-CN, et al Active-passive gradient shielding for MRI acoustic noise reduction. Magn Reson Med. Wiley Subscription Services, Inc., A Wiley Company; 2005;53:1013–7. 10.1002/mrm.20472 15844137

[14] Edelstein WA, Hedeen RA, Mallozzi RP, El-Hamamsy SA, Ackermann RA, Havens TJ. Making MRI quieter. Magn Reson Imaging. 2002;20:155–63. 12034336 10.1016/s0730-725x(02)00475-7

[15] Cho ZH, Chung ST, Chung JY, Park SH, Kim JS, Moon CH, et al A new silent magnetic resonance imaging using a rotating DC gradient. Magn Reson Med. 1998;39:317–21. 9469717 10.1002/mrm.1910390221

[16] Hennel F, Girard F, Loenneker T. “Silent” MRI with soft gradient pulses. Magn Reson Med. 1999;42:6–10. 10398943 10.1002/(sici)1522-2594(199907)42:1<6::aid-mrm2>3.0.co;2-d

[17] DEZWART J, VANGELDEREN P, Kellman P, DUYN J. Reduction of Gradient Acoustic Noise in MRI Using SENSE-EPI. Neuroimage. Academic Press; 2002;16:1151–5. 12202101 10.1006/nimg.2002.1119

[18] Setsompop K, Gagoski BA, Polimeni JR, Witzel T, Wedeen VJ, Wald LL. Blipped-controlled aliasing in parallel imaging for simultaneous multislice echo planar imaging with reduced g-factor penalty. Magn Reson Med. Wiley Subscription Services, Inc., A Wiley Company; 2012;67:1210–24. 10.1002/mrm.23097 21858868 PMC3323676

[19] Ireland CM, Giaquinto RO, Loew W, Tkach JA, Pratt RG, Kline-Fath BM, et al A novel acoustically quiet coil for neonatal MRI system. Concepts in Magnetic Resonance Part B: Magnetic Resonance Engineering. 2015;45:107–14. 10.1002/cmr.b.21287 26457072 PMC4594852

[20] Mansfield P, Haywood B, Coxon R. Active acoustic control in gradient coils for MRI. Magn Reson Med. Wiley-Blackwell; 2001;46:807–18. 11590659 10.1002/mrm.1261

[21] Ott M, Blaimer M, Grodzki DM, Breuer FA, Roesch J, Dörfler A, et al Acoustic-noise-optimized diffusion-weighted imaging. MAGMA. 2015;28:511–21. 10.1007/s10334-015-0492-5 26092411

[22] Corcuera-Solano I, Doshi A, Pawha PS, Gui D, Gaddipati A, Tanenbaum L. Quiet PROPELLER MRI Techniques Match the Quality of Conventional PROPELLER Brain Imaging Techniques. American Journal of Neuroradiology. American Journal of Neuroradiology; 2015;36:1124–7. 10.3174/ajnr.A4235 25678482 PMC8013025

[23] Fischer S, Grodzki DM, Domschke M, Albrecht M, Bodelle B, Eichler K, et al Quiet MR sequences in clinical routine: initial experience in abdominal imaging. Radiol Med. 2017;122:194–203. 10.1007/s11547-016-0710-x 27896570

[24] Grodzki DM, Jakob PM, Heismann B. Ultrashort echo time imaging using pointwise encoding time reduction with radial acquisition (PETRA). Magn Reson Med. Wiley-Blackwell; 2011;67:510–8. 10.1002/mrm.23017 21721039

[25] Pierre EY, Grodzki D, Aandal G, Heismann B, Badve C, Gulani V, et al Parallel imaging-based reduction of acoustic noise for clinical magnetic resonance imaging. Invest Radiol. 2014;49:620–6. 10.1097/RLI.0000000000000062 24743588

[26] Hamernik RP, Qiu W, Davis B. The effects of the amplitude distribution of equal energy exposures on noise-induced hearing loss: The kurtosis metric. The Journal of the Acoustical Society of America. Acoustical Society of America; 2003;114:386–95. 12880050 10.1121/1.1582446

[27] Kujawa SG, Liberman MC. Adding Insult to Injury: Cochlear Nerve Degeneration after “Temporary” Noise-Induced Hearing Loss. J. Neurosci. Society for Neuroscience; 2009;29:14077–85. 10.1523/JNEUROSCI.2845-09.2009 PMC2812055 19906956

[28] Baker MA. Reduction of MRI acoustic noise achieved by manipulation of scan parameters–A study using veterinary MR sequences. Radiography. Elsevier; 2013;19:11–6.

[29] PhD MM, PhD FGS. Auditory Noise Associated With MR Procedures: A Review. J Magn Reson Imaging. Wiley-Blackwell; 2000;12:37–45. 10931563 10.1002/1522-2586(200007)12:1<37::aid-jmri5>3.0.co;2-i

[30] Ott M, Blaimer M, Breuer F, Grodzki D, Heismann B, Jakob P. Acoustic noise reduction in T 1- and proton-density-weighted turbo spin-echo imaging. MAGMA. 2016;29:5–15. 10.1007/s10334-015-0502-7 26490348

[31] Goerner FL, Clarke GD. Measuring signal‐to‐noise ratio in partially parallel imaging MRI. Medical Physics. Wiley-Blackwell; 2011;38:5049–57. 10.1118/1.3618730 21978049 PMC3170395

[32] Sinkkila L, Väisänen J, Vaisanen O, Hyttinen J. Effects of ROI Size on Correlation between ROISR and SNR. 14th Nordic-Baltic Conference on Biomedical Engineering and Medical Physics. Berlin, Heidelberg: Springer Berlin Heidelberg; 2008. pp. 327–30.

